# Development of an Empirically Derived Measure of Food Safety Culture in Restaurants

**DOI:** 10.1016/j.jfp.2023.100043

**Published:** 2023-01-18

**Authors:** Adam Kramer, E. Rickamer Hoover, Nicole Hedeen, Lauren DiPrete, Joyce Tuttle, DJ Irving, Brendalee Viveiros, David Nicholas, Jo Ann Monroy, Erin Moritz, Laura Brown

**Affiliations:** 1 National Center for Environmental Health, U.S. Centers for Disease Control and Prevention, Atlanta, GA 30341, USA; 2 Minnesota Department of Health, USA; 3 Southern Nevada Health District, Las Vegas, Nevada, USA; 4 California Department of Health, California, USA; 5 Tennessee Department of Health, USA; 6 Rhode Island Department of Health, USA; 7 New York State Department of Health, USA; 8 Department of Epidemiology & Biostatistics, School of Public Health, University at Albany (SUNY), Rensselaer, New York. USA; 9 Harris County Department of Health, Houston, Texas, USA

**Keywords:** Food safety culture, Food safety, Food worker, Health knowledge attitudes practice, Restaurant

## Abstract

A poor food safety culture has been described as an emerging risk factor for foodborne illness outbreaks, yet there has been little research on this topic in the retail food industry. The purpose of this study was to identify and validate conceptual domains around food safety culture and develop an assessment tool that can be used to assess food workers’ perceptions of their restaurant’s food safety culture. The study, conducted from March 2018 through March 2019, surveyed restaurant food workers for their level of agreement with 28 statements. We received 579 responses from 331 restaurants spread across eight different health department jurisdictions. Factor analysis and structural equation modeling supported a model composed of four primary constructs. The highest rated construct was Resource Availability ($\bar{x}=4.69$, sd=0.57), which assessed the availability of resources to maintain good hand hygiene. The second highest rated construct was Employee Commitment ($\bar{x}=4.49$, sd=0.62), which assessed workers’ perceptions of their coworkers’ commitment to food safety. The last two constructs were related to management. Leadership ($\bar{x}=4.28$, sd=0.69) assessed the existence of food safety policies, training, and information sharing. Management Commitment ($\bar{x}=3.94$, sd=1.05)assessed whether food safety was a priority in practice. Finally, the model revealed one higher-order construct, Worker Beliefs about Food Safety Culture ($\bar{x}=4.35$, sd=0.53). The findings from this study can support efforts by the restaurant industry, food safety researchers, and health departments to examine the influence and effects of food safety culture within restaurants.

The Centers for Disease Control and Prevention (CDC) estimates that 48 million cases of domestically acquired foodborne illness occur annually in the United States, resulting in 325,000 hospitalizations and 3,000 deaths (Scallan, Griffin et al., 2011; Scallan, Hoekstra et al., 2011). Most reported foodborne illness outbreaks are attributed to restaurants (Dewey-Mattia et al., 2018). Past interventions to reduce foodborne illness have focused on addressing commonly identified risk factors associated with foodborne illness, such as ensuring food is cooked to recommended cooking temperatures and preventing contamination of the food (Olsen et al., 2000). Despite these important interventions, foodborne illnesses continue to occur. To further reduce the occurrence of foodborne outbreaks, Griffith et al. (2010b) proposed examining food safety culture as an emerging risk factor for foodborne illness.

Researchers (Griffith, Livesey, & Clayton, 2010b; Yiannas, 2008) have proposed varying definitions of food safety culture. The Global Food Safety Initiative, for example, defines food safety culture as “shared values, beliefs and norms that affect mind-set and behavior toward food safety in, across and throughout an organization” (Global Food, 2018). All the published definitions of food safety culture share a common element – that food workers’ shared beliefs influence food safety behavior.

Drawing from the organizational and safety culture literature, Griffith et al. (2010a) proposed that food safety culture was composed of five separate theoretical concepts related to food safety: (1) leadership, (2) communication, (3) commitment, (4) environment, and (5) risk awareness. This conceptualization focused primarily on the organizational factors thought to contribute to food safety culture.

Several studies have surveyed workers in a variety of settings in an attempt to develop a food safety culture assessment model. These researchers have assessed meat workers (Ball et al., 2010), culinary students (Neal et al., 2012), school food service workers (Abidin et al., 2014), and workers in a European meat distribution company (De Boeck et al., 2015). Taha et al. (2020) examined organizational factors and worker beliefs in food manufacturing plants. These studies identified anywhere from two to six separate theoretical concepts related to food safety culture among these populations. They found that beliefs about commitment (management and employee), resources (or infrastructure), and work pressures play a role in food safety culture.

Observing that much of the food safety culture research has been performed in food manufacturing facilities, the Environmental Health Specialists Network (EHS-Net) embarked on a study to develop a food worker survey measure that can be used to assess restaurant food safety culture at a specific time (sometimes referred to as the food safety climate (De Boeck et al., 2015). This paper reports on our development of this measure. EHS-Net is a collaborative network of the CDC, the U.S. Food and Drug Administration, the U.S. Department of Agriculture’s Food Safety and Inspection Service, and eight health departments. A CDC cooperative agreement funded health departments in California, Harris County (TX), Minnesota, New York, New York City (NY), Rhode Island, the Southern Nevada Health District, and Tennessee to participate in EHS-Net and in this study.

## Methods

### Survey development

We developed a survey for restaurant food workers based on the constructs proposed by Griffith et al. (2010a) and previously administered surveys (Ball et al., 2010; De Boeck et al., 2015; Neal et al., 2012). A workgroup composed of EHS-Net health department staff designed the survey to apply to all types of restaurants, rather than for a specific company as previous researchers have done (i.e., our survey assessed handwashing resources, something that is relevant in all restaurants). The survey asked food workers to self-report their level of agreement with 28 statements (Table 2) using a Likert-scale ranging from 1 (strongly disagree) to 5 (strongly agree). Four of these items were reverse-coded (DeVellis, 2003). Table 2 includes the survey items, the number of responses, the mean scores (higher scores show stronger agreement [or disagreement for reverse-coded items]), and standard deviations. We also included questions to assess food workers’ food safety knowledge and experience working in restaurant kitchens.

To increase restaurant participation in the study and food worker honesty in the survey responses, study data collection was anonymous. Thus, to ensure we did not collect data that could allow the identification of food workers, we asked limited questions about individual demographics. For example, we did not collect data on staff race, ethnicity, or age.

The instrument was pilot tested with three restaurant food workers for comprehension and length of time to complete the survey. The survey was translated into Spanish by one native speaker and translated back into English by another native speaker to verify the translation. A copy of the food worker survey is provided in the Supplementary material, and all the study materials are posted at https://www.cdc.gov/nceh/ehs/ehsnet/study_tools/index.htm.

### Sample

The study sample consisted of randomly selected restaurants in each of the eight EHS-Net health department’s jurisdictions. Restaurants were defined as establishments that prepare and serve food or beverages to customers but are not institutions, food carts, mobile food units, temporary food stands, supermarkets, restaurants in supermarkets, or caterers. In each EHS-Net jurisdiction, staff chose a geographic area in which to recruit restaurants for study participation, based on a reasonable travel distance (mean=88.1 min, range = 30 min to 4 h). One jurisdiction was urban; the other seven were a combination of urban, suburban, and rural areas. The staff then sent a list of restaurants within that area to CDC, which selected a random sample of restaurants for each jurisdiction. Staff in each jurisdiction requested voluntary study participation from managers in a random sample of restaurants and scheduled a data collection visit through telephone calls or visits to the participating restaurants. Within each restaurant, food workers were requested to voluntarily participate in the study. No incentives were provided to participate in this study.

### Data collection

Data collection took place from March 2018 to March 2019. Data were collected by EHS-Net staff. All data collectors participated in training designed to promote data collection consistency. At each restaurant, data collectors, EHS-Net staff, interviewed a manager (someone who had authority over the restaurant) about restaurant characteristics, asked food workers (staff members who prepare food in the restaurant) to complete a survey, and conducted an observation of food preparation and storage practices in the kitchen area. This paper presents data from the food worker survey on food workers’ beliefs about food safety.

Food workers completed a self-administered survey about their beliefs around food safety in their restaurant. The survey was provided in English and Spanish using the SurveyMonkey (Momentive, San Mateo, CA, USA) online survey platform. Food workers could either complete the survey online using the online application (at their convenience) or complete a paper version of the survey during the data collection visit. Any surveys completed on paper forms were entered into SurveyMonkey later by the data collectors. We did not record whether a food worker completed an electronic or paper-based survey.

A study identifier was used to link worker survey data to the matching restaurant; however, we did not collect data that could identify individual restaurants, managers, or workers. Each EHS-Net jurisdiction’s institutional review board approved the study protocol.

### Analysis

We randomly split the completed survey responses into two groups (*n* = 248 per group) for analysis. One group was used for model building, and the other group was used for validation of the statistical model. We examined the model fit of the theoretical model of food safety culture based on the constructs proposed by Griffith et al. (2010a) using confirmatory factor analysis. To assess fit, we examined the overall concordance of multiple indices; these included the chi-square (not statistically significant indicated a better fit), the standardized root mean square residual (SRMR ≤ 0.08 indicates a better fit), the comparative fit index (CFI ≥ 0.95 indicates a better fit), and root mean square error of approximation (RMSEA < 0.06 indicates a better fit) (Schreiber et al., 2006). However, the data that we collected did not support this model structure. Therefore, we then conducted an exploratory factor analysis to identify the factors that were empirically supported. We retained items that loaded onto unique common factors, had a primary factor loading of 0.4 or above, and did not load onto another factor at 0.3 or above. We then examined whether the data would benefit from a data reduction method using Bartlett’s test of sphericity, where a significant value supports further data reduction. We then examined the number of factors that would be supported by the model using a scree test and minimum average partials test (Velicer et al., 2000). Once we identified the four factors and their associated items, we assessed scale reliability using Cronbach’s alpha with an alpha coefficient of 0.65 or higher considered acceptable. After that, we conducted structural equation modeling to identify an appropriate model structure and to determine if the data would support further generalization to a higher-order factor. Finally, we created a composite measure for each factor in the model, based on the structural equation model, where the sum of the Likert-scaled questions for each factor was calculated. Negatively phrased questions were recoded so that higher scores would equate to positive agreement (e.g., strong disagreement with a negatively phrased item was recoded as strong agreement for analysis). We then divided the sum by the number of questions associated with the factor to provide a standardized score for each factor. We used SAS version 9.4 (SAS Institute, Cary, NC, USA) to analyze the data.

## Results

### Demographics

We contacted 1,496 restaurants to participate in the study, of which 506 were excluded (restaurants were no longer in business, were not a restaurant [e.g., a grocery store], the manager was unable to communicate with the study recruiting staff in English). Of the remaining 990 restaurants, we had participation from 331 restaurants. We received 579 food worker survey responses from those 331 different restaurants. Manager interview data indicated that the study restaurants were largely independently owned (57.1%). Food worker survey data showed that the largest group of food workers had 1–5 years of experience (39.9%), 1–5 years of tenure in their current establishment (46.6%), and worked primarily in the kitchen (55.4%). Most respondents had a current Certified Food Protection Manager credential (56.7%); however, only 9.5% were in a supervisory role. Food workers reported primarily speaking English (72.5%) and Spanish (18%). Most of the food workers had completed high school (32.5%), had at least some college education (49.1%), and were male (50.6%) (Table 1).

### Data screening

Of the 579 responses, 44 (7.6%) were completed in Spanish. All Likert-scaled items were initially tested for multicollinearity, deviation from linearity, consistency with similar items, and if all items were answered. This screening led us to drop item 5 (Table 2) from further analyses because it did not consistently correlate with other similar questions. This lack of correlation is likely due to the influence of varied glove use requirements across the jurisdictions.

### Theoretical model

An initial theoretical model based on Griffith et al. (2010a) work was constructed where we associated each of the items to one of five constructs: Commitment; Communication; Leadership; Resources; and Risk Awareness. We then assessed this model for fit. However, our data did not support this model. None of the fit indices — *χ*^2^(485) = 2,039.45, *p* < 0.0001; SRMR = 0.10; CFI = 0.68; and RMSEA = 0.11 (0.12, 0.11) — indicated an adequate fit between the data and the model.

### Exploratory factor analysis

We initially included 27 items in a factor analysis. Sixteen questions were retained in the model. The principal factor analysis used squared multiple correlations with all other items, unweighted least squares factors, and a promax (oblique) rotation. The remaining 11 items did not load onto common factors or meet the above criteria for retention in further analyses.

A significant Bartlett’s test of sphericity (*χ*^2^[62] = 133.92, *p* < 0.0001) indicated that the data could be reduced into factors. Results of a scree test and a minimum average partials test suggested four factors would be sufficient to explain the variance (Velicer et al., 2000).

Table 3 shows the survey items and factor loadings. The EHS-Net working group reviewed the items that formed each of the four factors to provide their perceptions of the constructs measured by each factor. The working group labeled those factors as Leadership, Management Commitment, Employee Commitment, and Resource Availability. Leadership included six items, Employee Commitment included four items, and Resource Availability and Management Commitment included three items each.

Scale reliability for each factor was assessed using Cronbach’s alpha; each factor had acceptable reliability (Leadership = 0.88, Employee Commitment = 0.87, Resource Availability = 0.72, Management Commitment = 0.73) (Nunnally, 1978). External validity was assessed using the reserved half of the dataset; the results were similar to those obtained from the first half of the data.

### Structural equation modeling

Structural equation modeling was used to identify the relationships among the factors and to determine if the data would support a higher-order factor (Anderson and Gerbing, 1988). In other words, this modeling was to determine if the identified factors are stand-alone factors, are inter-related, and if they can be further generalized to a higher-order factor (an overarching factor that is explained by these primary factors, similar to how individual questions explain the primary factors).

**Figure 1.**
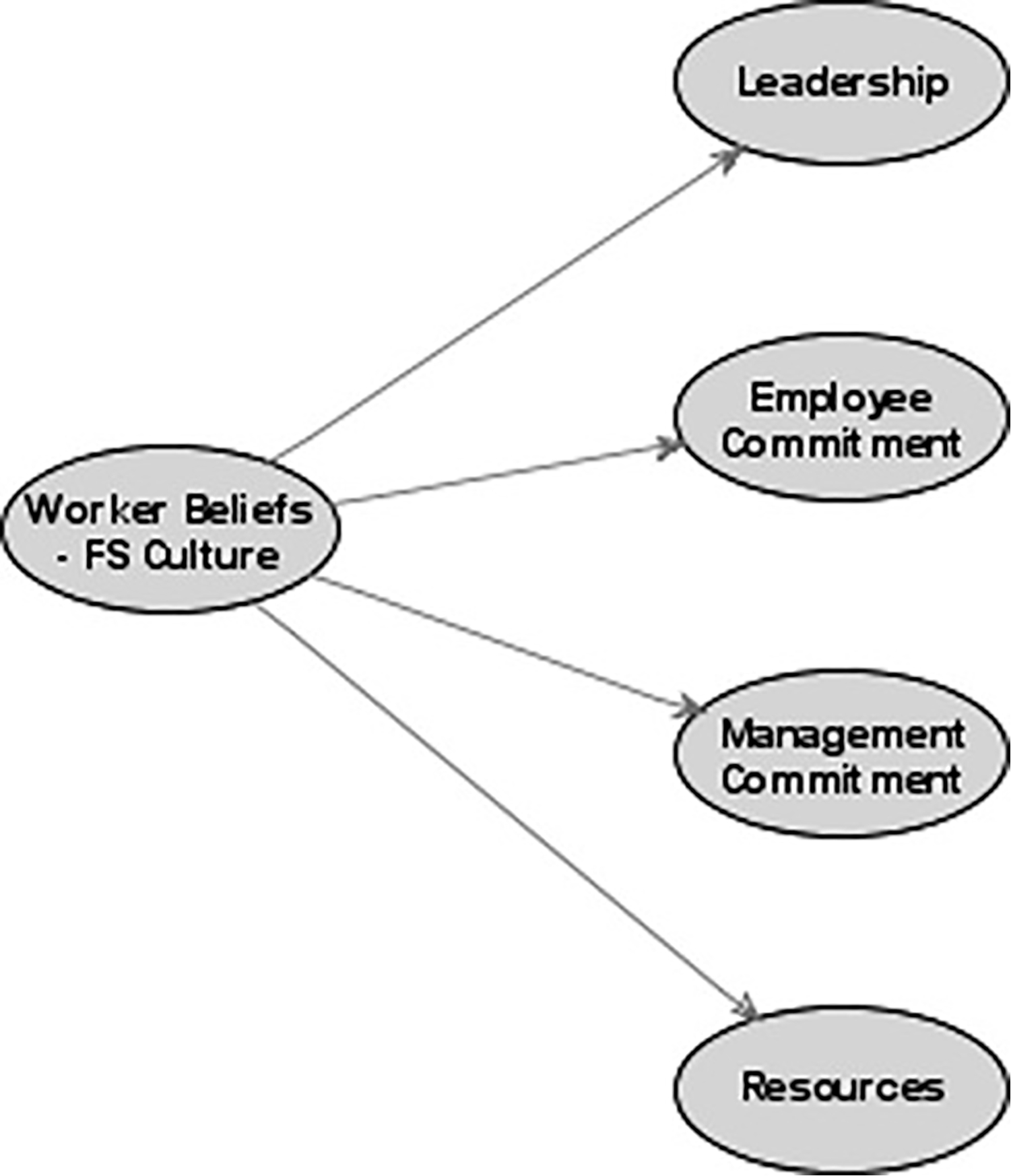
Path diagram of food safety culture model.

We examined various structural forms of the factors identified in the exploratory factor analysis; a model with one higher-order factor (Worker beliefs about food safety culture) was found to be optimal. The fit indices for this model showed an overall good fit — *χ*^2^(100) = 210.78, *p* < 0.0001; SRMR = 0.05; CFI = 0.95; and RMSEA = 0.07 (0.08,0.05) (Figure 1).

Reliability estimates were generally acceptable (Table 4). Item reliability was generally above 0.5, except for item 7 (0.27). We chose to retain this item because of its contextual similarity to other items and to maintain factor reliability. The four primary factors exhibited acceptable overall composite reliability (Leadership = 0.91, Employee Commitment = 0.89, Resource Availability = 0.79, and Management Commitment = 0.78). Relationships between individual items and their associated factor were examined; all pathways were significant. Similarly, the relationships between each of the primary factors were significantly associated with the higher-order factor (Table 4). The finding that the *t*-values are significant for these path coefficients suggests that the items are measuring the same construct.

### Scale measures

All constructs had composite scores spanning the entire range, from 1 (strongly disagree) to 5 (strongly agree). In general, food workers viewed each of the factors positively (composite score >3), although individual workers in some restaurants reported lower scores. Food workers viewed Resource Availability highest (mean = 4.69, SD = 0.57), followed by Employee Commitment (mean = 4.49, SD = 0.62), Leadership (mean = 4.28, SD = 0.69), and Management Commitment (mean = 3.94, SD = 1.05). The overall belief in food safety culture had a mean score of 4.35 (SD = 0.53) (Table 4).

## Discussion

Our intent for this study was to provide convergent validity in support of existing food safety culture models within restaurant food workers. Because our data did not support the application of any of the existing published models of food safety culture, we created a new model. Our model is not wholly unique and does share some common factors with previously published models. Similar to other models, we identified a Leadership factor (Abidin, Fatimah, Arendt, & Strohbehn, 2014; De Boeck, Jacxsens, Bollaerts, & Vlerick, 2015; Griffith, Livesey, & Clayton, 2010a; Taha, Wilkins, Juusola, & Osaili, 2020) and Resources factor (Abidin et al., 2014; De Boeck et al., 2015). However, while some researchers have identified a single construct of commitment (Abidin, Fatimah, Arendt, & Strohbehn, 2014; De Boeck, Jacxsens, Bollaerts, & Vlerick, 2015; Griffith, Livesey, & Clayton, 2010a), we found two commitment-related constructs – one for managers and one for workers (Ball et al., 2010; Neal et al., 2012; Taha et al., 2020). Additionally, other researchers have identified constructs which our data did not support, such as risk awareness (Abidin, Fatimah, Arendt, & Strohbehn, 2014; De Boeck, Jacxsens, Bollaerts, & Vlerick, 2015; Griffith, Livesey, & Clayton, 2010a). Differences between our model and others may be because food safety culture constructs differ across settings (Abidin et al., 2014; Ball et al., 2010; De Boeck et al., 2015; Neal et al., 2012; Taha et al., 2020). Our findings might also be the result of our sample being comprised of a large and heterogenous (331 restaurants spread across eight different jurisdictions) sample compared with the limited sampling frames available to other researchers.

The items making up Resource Availability, the construct with the highest rated composite score of the four, assess the availability of resources needed to maintain good hand hygiene. This high score was not unexpected; hand hygiene resources are a basic component of food safety and are assessed during inspections.

The items making up Employee Commitment assess workers’ perceptions of their coworkers’ commitment to food safety (e.g., employees follow food safety rules even when no one is looking). This construct was relatively highly rated, suggesting that workers in our study believed their coworkers were committed to food safety. Employee commitment to food safety likely leads to social norms that are supportive of food safety behavior; social norms can be important predictors of behavior (Yiannas, 2008).

Two of the unique constructs directly tied to management: Leadership and Management Commitment. Leadership deals primarily with stated food safety policies, training, and information sharing (questions such as: The restaurant provides sufficient food safety training for me to do my job). Management Commitment covers prioritizing food safety practice (with questions such as: When the restaurant is busy, managers prioritize serving food over following food safety rules). These constructs had the lowest overall scores and highest variation in scores. This dichotomy might result from the difference between the stated practices (Leadership) and their implementation (Management Commitment). We take this to mean that restaurants might have good practices in place, but the pragmatic realities of operating a restaurant might result in lapses in the application of those practices.

We were able to further generalize the results of this study to a higher-order construct composed of the results of the four primary constructs. This higher-order construct provides a high-level view of the overall food safety culture in a restaurant. This finding also indicates that food safety culture may be a part of the larger organizational culture in the restaurant.

We also assessed risk awareness (e.g., If food safety rules are not followed, a customer might become sick). However, these questions did not load onto a unique factor, suggesting that restaurant food workers might have highly variable views of the risk posed by food. This finding suggests that perceptions of risk may be less important to food safety culture than manager and worker commitment to specific food safety behaviors.

This study has at least six limitations. First, the survey was self-administered, which would require the food worker to be able to read. Second, the survey was provided only in English and Spanish, which required the food worker to comprehend one of these languages to complete the survey. The potential universe of primary languages used by food workers is likely much greater than these two languages. Third, because the survey responses were self-reported, responses are subject to social desirability bias, which might have resulted in overreporting of socially desirable responses, such as positive views of food safety. Fourth, since limited information was collected about the food workers’ individual characteristics, we are unsure of the comparability of our sample to all food workers. Fifth, responses were from voluntarily participating restaurants. Responses from restaurants that did not participate might have differed, leading to a potential selection bias. Finally, because turnover is high in the restaurant industry (National Restaurant Association, 2014), worker beliefs about food safety culture captured at the time of our study might not be representative of worker beliefs in restaurants over time.

We have provided a new, empirically derived model for assessing worker’s beliefs about food safety culture. This model is based on restaurant workers’ level of agreement with statements about the food safety within their restaurant. Restaurants can use this tool to obtain a benchmark of their workers’ views of food safety. The tool can also be used to assess changes in perceptions of food safety over time and the effect of interventions designed to improve the food safety culture.

This model could be further refined. Eleven of the questions we asked did not load onto any constructs. This might be because of additional constructs that we did not ask about (such as work pressures or worker burnout). We recommend further evaluation and refinement of the questions to determine if there are food safety culture factors our study did not assess. Further, we recommend developing additional questions around the existing factors that we identified. This will serve to strengthen the factors (from a statistical standpoint) and allow researchers to more narrowly define what the constructs are measuring.

## Supplementary Material

Survey instrument

## Figures and Tables

**Table 1:** Respondent demographics

Demographic characteristic	*N*	Percentage
Restaurant ownership		
Independently owned	189	57.1
Chain owned	138	41.7
Not reported	4	1.2
Years of experience in food service		
<1	54	9.3
1–5	231	39.9
6–10	110	19
11–15	60	10.4
>15	103	17.8
Not reported	21	3.6
Years’ tenure in the current restaurant		
<1	191	33
1–5	270	46.6
6–10	65	11.2
11–15	22	3.8
>15	22	3.8
Not reported	9	1.6
Certified food protection manager		
Currently certified	328	56.7
Previously certified	54	9.3
Not certified	182	31.4
Not Reported	15	2.6
Primary area of the restaurant that they work in		
Kitchen/food preparation	321	55.4
Food service/bar	155	26.8
Management	55	9.5
Other	35	6.1
Not reported	13	2.3
Sex		
Male	293	50.6
Female	260	44.9
Not reported	26	4.5
Self-reported primary language		
English	420	72.5
Spanish	104	18
Chinese	16	2.8
Other	26	4.5
Not reported	13	2.3
Level of formal education		
Less than High school graduate	73	12.6
High school graduate	188	32.5
Post high school	284	49.1
Not reported	34	5.9

**Table 2:** Descriptive data on the Food Safety Culture Tool items

Item	*N* ^a^	Mean^b^	SD
1. Employees follow food safety rules, even when no one is looking	578	4.45	0.74
2. Employees encourage each other to follow food safety rules	578	4.43	0.79
3. Employees take responsibility for food safety in their areas	578	4.51	0.70
4. Employees wash their hands when they are supposed to	576	4.56	0.69
5. Employees touch food that will not be cooked with their bare hands **(Reverse coded)**^c^	571	2.09	1.37
6. Employees do not work while they are sick with vomiting or diarrhea	577	4.38	1.12
7. There are enough gloves or utensils to use to avoid touching the food with my bare hands	577	4.57	0.96
8. Sinks are nearby and are easy to get to for handwashing	576	4.74	0.57
9. Sinks for handwashing have hot water, soap, and paper towels or another way to dry my hands	578	4.77	0.56
10. Equipment is well maintained and operates properly	578	4.45	0.81
11. There is enough staff to cover when the restaurant is busy	576	4.12	1.00
12. There is enough staff to cover when an employee does not come into work	577	3.89	1.08
13. Employees have to cut corners because there is too much work to do **(Reverse coded)**	573	3.89	1.22
14. Managers encourage employees to follow food safety rules	576	4.64	0.71
15. When the restaurant is busy, managers prioritize serving food over following food safety rules **(Reverse coded)**	570	3.67	1.48
16. Managers encourage employees to report food safety problems	577	4.44	0.83
17. Managers ignore when employees are not following food safety rules **(Reverse coded)**	576	4.22	1.20
18. Managers are aware of the food safety rules	573	4.64	0.71
19. Managers strive to improve food safety practices	563	4.51	0.71
20. If food safety rules are not followed a customer may become sick	575	4.62	0.73
21. The restaurant provides sufficient food safety training for me to do my job	578	4.43	0.82
22. I know what the food safety rules are for my job	575	4.65	0.61
23. Food safety is stressed with signs, posters, or in-shift meetings	571	4.22	1.00
24. Employees are positively recognized for following food safety rules	574	4.00	1.04
25. Managers get feedback from employees to improve food safety	574	4.02	1.00
26. Employees know the restaurant’s food safety expectations	574	4.47	0.68
27. My manager explains what is expected of me	575	4.53	0.69
28. It is easy to talk with my manager about any problems	576	4.45	0.89

a Respondents were not required to answer every question resulting in varying response rates.

b Scores can range from 1 (strongly disagree) to 5 (strongly agree). Higher scores indicate stronger agreement with the statement or disagreement for reverse-coded items.

c Question 5 was dropped from the analysis because it was not consistently correlated with other similar questions.

**Table 3:** Factor loadings and communalities based on factor analysis with promax rotation for 16 items from the Food Safety Culture Survey Tool (*n* = 248)

Item	Factor 1 - Leadership	Factor 2 - Employee Commitment	Factor 3 - Resources	Factor 4 - Management Commitment	Communality
21. The restaurant provides sufficient food safety training for me to do my job	0.82				0.72
25. Managers get feedback from employees to improve food safety	0.79				0.65
23. Food safety is stressed with signs, posters, or in-shift meetings	0.76				0.55
24. Employees are positively recognized for following food safety rules	0.73				0.58
27. My manager explains what is expected of me	0.60				0.66
26. Employees know the restaurant’s food safety expectations	0.58				0.64
3. Employees take responsibility for food safety in their areas		0.82			0.78
2. Employees encourage each other to follow food safety rules		0.80			0.70
1. Employees follow food safety rules, even when no one is looking		0.71			0.69
4. Employees wash their hands when they are supposed to		0.55			0.53
8. Sinks are nearby and are easy to get to for handwashing			0.85		0.75
9. Sinks for handwashing have hot water, soap, and paper towels or another way to dry my hands			0.77		0.64
7. There are enough gloves or utensils to use to avoid touching the food with my bare hands			0.56		0.29
15. When the restaurant is busy, managers prioritize serving food over following food safety rules **(Reverse coded)**				0.81	0.62
13. Employees have to cut corners because there is too much work to do **(Reverse coded)**				0.72	0.56
17. Managers ignore when employees are not following food safety rules **(Reverse coded)**				0.68	0.49

*Note*: Factor loadings <0.3 are suppressed.

**Table 4:** Properties of the Food Safety Culture Structural Equation Model

Constructs and Items	Standardized loading	*t* ^a^	Reliability	Variance extracted estimate	Mean	Standard Deviation
**Leadership**			0.91^b^	0.38	4.28	0.69
21. The restaurant provides sufficient food safety training for me to do my job	0.83	35.98	0.69			
23. Food safety is stressed with signs, posters, or in-shift meetings	0.73	22.11	0.53			
24. Employees are positively recognized for following food safety rules	0.75	23.58	0.55			
25. Managers get feedback from employees to improve food safety	0.77	26.12	0.59			
26. Employees know the restaurant’s food safety expectations	0.81	31.72	0.65			
27. My manager explains what is expected of me	0.81	32.17	0.66			
**Employee commitment**			0.89^b^	0.37	4.49	0.62
1. Employees follow food safety rules, even when no one is looking	0.84	35.46	0.70			
2. Employees encourage each other to follow food safety rules	0.82	32.86	0.67			
3. Employees take responsibility for food safety in their areas	0.88	44.02	0.77			
4. Employees wash their hands when they are supposed to	0.73	21.89	0.53			
**Resources**			0.79^b^	0.32	4.69	0.57
7. There are enough gloves or utensils to use to avoid touching the food with my bare hands	0.52	9.95	0.27			
8. Sinks are nearby and are easy to get to for handwashing	0.88	26.88	0.77			
9. Sinks for handwashing have hot water, soap, and paper towels or another way to dry my hands	0.80	22.89	0.64			
**Management commitment**			0.78^b^	0.32	3.94	1.05
13. Employees have to cut corners because there is too much work to do **(Reverse coded)**	0.76	18.02	0.58			
15. When the restaurant is busy, managers prioritize serving food over following food safety rules **(Reverse coded)**	0.74	16.95	0.55			
17. Managers ignore when employees are not following food safety rules **(Reverse coded)**	0.71	15.72	0.51			
**Workers’ beliefs about food safety culture**					4.35	0.53

a *t* tests assessed the pathways between all items and the constructs. All *t* tests were significant at *p* < 0.0001.

b Denotes composite reliability.
