# Supplementary material for: Development of an Empirically Derived Measure of Food Safety Culture in Restaurants
Source: J Food Prot. Author manuscript; Available in PMC 2023 Mar 16. (PMC10018426; doi:10.1016/j.jfp.2023.100043)
Supplement: Survey instrument [file NIHMS1874535-supplement-Survey_instrument.docx]

**Supplementary Material – Food worker survey**

This supplement has the food worker survey form that was voluntarily filled out by food workers in the restaurants participating in this study. Verbal consent was obtained from the participants before they completed the survey. The complete study protocol and materials are available at <https://www.cdc.gov/nceh/ehs/ehsnet/study_tools/index.htm>.

FOOD SAFETY PRACTICES

Please indicate how much you agree with the following statements based on your experiences in this restaurant. These responses cannot be linked back to you, so please be as honest as possible.

|  |  | Strongly Disagree | Disagree | Neither Agree nor Disagree | Agree | Strongly Agree |
| --- | --- | --- | --- | --- | --- | --- |
|  | Employees follow food safety rules, even when no one is looking | 1 | 2 | 3 | 4 | 5 |
|  | Employees encourage each other to follow food safety rules | 1 | 2 | 3 | 4 | 5 |
|  | Employees take responsibility for food safety in their areas | 1 | 2 | 3 | 4 | 5 |
|  | Employees wash their hands when they are supposed to | 1 | 2 | 3 | 4 | 5 |
|  | Employees touch food that will not be cooked with their bare hands | 1 | 2 | 3 | 4 | 5 |
|  | Employees do not work while they are sick with vomiting or diarrhea | 1 | 2 | 3 | 4 | 5 |
|  | There are enough gloves or utensils to use to avoid touching the food with my bare hands | 1 | 2 | 3 | 4 | 5 |
|  | Sinks are nearby and are easy to get to for handwashing | 1 | 2 | 3 | 4 | 5 |
|  | Sinks for handwashing have hot water, soap, and paper towels or another way to dry my hands | 1 | 2 | 3 | 4 | 5 |
|  | Equipment is well maintained and operates properly | 1 | 2 | 3 | 4 | 5 |
|  | There is enough staff to cover when the restaurant is busy | 1 | 2 | 3 | 4 | 5 |
|  | There is enough staff to cover when an employee does not come in to work | 1 | 2 | 3 | 4 | 5 |
|  | Employees have to cut corners because there is too much work to do | 1 | 2 | 3 | 4 | 5 |
|  | Managers encourage employees to follow food safety rules | 1 | 2 | 3 | 4 | 5 |
|  | When the restaurant is busy, managers prioritize serving food over following food safety rules | 1 | 2 | 3 | 4 | 5 |
|  | Managers encourage employees to report food safety problems | 1 | 2 | 3 | 4 | 5 |
|  | Managers ignore when employees are not following food safety rules | 1 | 2 | 3 | 4 | 5 |
|  | Managers are aware of the food safety rules | 1 | 2 | 3 | 4 | 5 |
|  | Managers strive to improve food safety practices | 1 | 2 | 3 | 4 | 5 |
|  |  | Strongly Disagree | Disagree | Neither Agree nor Disagree | Agree | Strongly Agree |
|  | If food safety rules are not followed a customer may become sick | 1 | 2 | 3 | 4 | 5 |
|  | The restaurant provides sufficient food safety training for me to do my job | 1 | 2 | 3 | 4 | 5 |
|  | I know what the food safety rules are for my job | 1 | 2 | 3 | 4 | 5 |
|  | Food safety is stressed with signs, posters, or in shift meetings | 1 | 2 | 3 | 4 | 5 |
|  | Employees are positively recognized for following food safety rules | 1 | 2 | 3 | 4 | 5 |
|  | Managers get feedback from employees to improve food safety | 1 | 2 | 3 | 4 | 5 |
|  | Employees know the restaurant’s food safety expectations | 1 | 2 | 3 | 4 | 5 |
|  | My manager explains what is expected of me | 1 | 2 | 3 | 4 | 5 |
|  | It is easy to talk with my manager about any problems | 1 | 2 | 3 | 4 | 5 |
|  | Unsafe food always smells or looks bad | 1 | 2 | 3 | 4 | 5 |
|  | It is okay to eat undercooked or raw meats as long as they are organic | 1 | 2 | 3 | 4 | 5 |
|  | It is okay to eat undercooked or raw meats as long as they are local | 1 | 2 | 3 | 4 | 5 |
|  | Eating raw or undercooked meats increases your risk for illness | 1 | 2 | 3 | 4 | 5 |
|  | Food that has been cooked and then refrigerated can continue to be used until it looks or smells bad | 1 | 2 | 3 | 4 | 5 |
|  | Once cooked, food no longer has any bacteria on it | 1 | 2 | 3 | 4 | 5 |

1. The warning statements on some menus about eating raw or undercooked food is there because (check all that apply):

☐ The government requires it

☐ The restaurant is trying to avoid responsibility if someone gets sick

☐ The restaurant wants to let customers know which items may be riskier to eat

☐ I haven't seen a warning on any menus

☐ Other: _____________________________________________________________________

Please answer the next questions to the best of your knowledge. Some of the practices asked about may not occur in this restaurant.

1. Hands should be washed prior to handling food, plates or utensils after (check all that apply)

☐ They only need to be washed before handling food not before handling plates or utensils

☐ If an employee uses gloves they don't need to wash their hands

☐ Using the toilet

☐ Between handling raw and cooked foods

☐ Coughing or sneezing into their hand

1. Cold held meats or other refrigerated items should be kept at ____ or below (pick one)

☐ 32F (0C)

☐ 41F (5C)

☐ 45F (7C)

☐ 50F (10C)

1. Hot held meats or other hot items should be kept at ____ or higher (pick one)

☐ 120F (49C)

☐ 135F (57C)

☐ 140F (60C)

☐ 150F (66C)

1. What symptom(s) are most likely to show that an employee has an illness that can be passed through food (pick one)

☐ Pink eye

☐ Runny nose and sneezing

☐ Vomiting and diarrhea

☐ All of the above

1. What is the proper procedure for washing your hands (pick one)

☐ Wet hands with warm water. Apply soap. Vigorously scrub hands and arms for 10-20 seconds. Rinse hands. Dry hands.

☐ Wet hands with warm water. Apply soap. Vigorously scrub hands and arms for 2-5 seconds. Apply a hand antiseptic (such as hand santizer.) Dry hands.

☐ Wet hands with warm water. Apply soap. Rinse hands. Dry hands.

☐ None of the above

1. Food that is cooked and then cooled must be cooled down within the following timeframe (pick one):

☐ 135F/140F to 70F within 2 hours and then reach 41F/45F within an additional 4 hours (6 hours total)

☐ 135F/140F to 70F within 4 hours and then reach 41F/45F within an additional 8 hours (12 hours total)

☐ It doesn’t matter if the food has been cooked thoroughly

☐ None of the above

1. The proper order for dishwashing (either manually in a three-compartment sink or using a dishwasher) is (pick one):

☐ Rinse, wash with soapy water, sanitize, air dry

☐ Wash with soapy water, rinse with clean water, sanitize, air dry

☐ Wash with soapy water, sanitize, towel dry

☐ None of the above

DEMOGRAPHIC / CLASSIFICATION

1. How many years have you worked in food service? [Check only one]

☐ Less than 1 year ☐1-5 years ☐ 6-10 years ☐ 11-15 years ☐ More than 15 years ☐ Prefer not to answer

1. Have you had any food safety training while employed at this restaurant?

☐ Yes ☐ No ☐ Prefer not to answer

1. Have you ever been certified in food safety?

☐ Yes ☐ No *If yes, is the certification still valid?* ☐ Yes ☐ No

1. Have you ever been a Certified Food Protection Manager (such as by passing an ANSI accredited program such as ServSafe, Prometric, National Registry of Food Safety Professionals, 360Training, or AboveTraining)?

☐ Yes ☐ No *If yes, is the certification still valid?* ☐ Yes ☐ No

1. How long have you been employed at this restaurant? [Check only one]

☐ Less than 1 year ☐1-5 years ☐ 6-10 years ☐ 11-15 years ☐ More than 15 years

1. What area of the kitchen do you primarily work in? [Check only one]

☐ Cook line ☐Food prep ☐ Cook line & Food prep ☐ Serving ☐ Bar ☐ Dishwashing ☐ Supervision ☐ Other:_____________________

1. What is your gender?

☐ Male ☐ Female ☐ Other ☐ Prefer not to answer

1. What is the highest level of formal education you have completed?

| ☐ 8^th^ grade or less | ☐ Community college or associate degree | ☐ Graduate degree |
| --- | --- | --- |
| ☐ Some high school | ☐ Some college | ☐ Other: __________________ |
| ☐ High school diploma/GED | ☐ Bachelors degree | ☐ Prefer not to answer |
| ☐ Some community college | ☐ Some graduate school |  |

1. Have you attended a culinary training or culinary arts program?

☐ Yes - Completed the program ☐ Yes - Have taken courses but did not complete the program or I am currently taking classes ☐ No

1. What is your primary language (the language that you speak best)?

| ☐ English | ☐ Chinese (any dialect) | ☐ Korean | ☐ Polish |
| --- | --- | --- | --- |
| ☐ Spanish | ☐ Japanese | ☐ German | ☐ Other:___________ |
| ☐ Russian | ☐ Vietnamese | ☐ Italian | ☐ Prefer not to answer |
